# Exploring oral health related awareness, perceptions, practices and experiences among type 2 diabetes mellitus patients: a mixed method design

**DOI:** 10.1186/s12903-025-06153-5

**Published:** 2025-05-24

**Authors:** Aymen Elsous, Aesha Fetaiha, Mahmoud Radwan

**Affiliations:** 1 Faculty of Medical Sciences, Israa University, Gaza Strip, Palestine; 2 Analysis and Decision Support Department, Unit of Planning and Institutional Performance Development, Ministry of Health, Gaza Strip, Palestine; 3 Medical Aids for Palestinians, Gaza Strip, Palestine; 4 Aesha Fetaiha Dental Clinic, Gaza Strip, Palestine; 5 General Directorate of International Cooperation and Projects, Foreign Relations, Ministry of Health, Gaza Strip, Palestine

**Keywords:** Knowledge, Attitudes, Practices, Experiences, Diabetes Mellitus, Oral health

## Abstract

**Background/Objective:**

The World Health Organization has classified diabetes mellitus as a pandemic disease, which is causing it to become a major worldwide health concern. It can have significant long-term repercussions, especially on dental health, if treatment is not received. The study aimed to explore patients with type 2 diabetes, awareness to, perceptions to and practices regarding oral health, in addition to their experiences.

**Materials and methods:**

Convergent triangulation was applied from May to November 2022. 376 patients with type 2 diabetes mellitus participated in the quantitative part and 13 patients involved in the qualitative study. The quantitative study was applied in five randomly selected primary health centers representing the five Gaza governorates using self-developed semi-structured questionnaire. The qualitative study was applied in two central primary health centers. Descriptive analysis was applied using the SPSS software and thematic analysis was approached for the qualitative study.

**Results:**

64.4% of patients see themselves susceptible to oral health problems, and 67.8% perceived severity of oral complications of diabetes mellitus. Moreover, 73.2% perceived benefits from oral health practices and 56.2% perceived barriers to oral health practices. Patients' awareness regarding oral health complications from DM and oral health practices are inadequate (57.6%), as well as oral health habits (42.5%). Main themes elucidated from the qualitative study are dental care service quality, patient-dentist interaction, oral hygiene and self-care, and the patient's experiences with oral health problems.

**Conclusions:**

The results point to important gaps in patients' knowledge and attitudes about dental health in relation to diabetes mellitus. Even though most people are aware of their vulnerability to oral health problems and the seriousness of any potential consequences, a sizable portion continue to believe that there are obstacles in the way of good oral hygiene habits. Poor oral hygiene practices and a lack of knowledge about oral health issues point to the need for more focused interventions and improved educational programs. The significance of high-quality dental care services, productive patient-dentist relationships, and encouraging self-care behaviors are all emphasized by the qualitative findings. In order to improve patient outcomes and encourage better dental hygiene habits among diabetics, these issues must be addressed. This will ultimately reduce oral health complications and increase general well-being.

**Supplementary Information:**

The online version contains supplementary material available at 10.1186/s12903-025-06153-5.

## Introduction

A metabolic endocrine ailment marked by hyperglycemia, diabetes mellitus (DM) is one of the chronic conditions with the fastest rate of growth in the globe [1]. With its alarmingly high incidence, diabetes mellitus is becoming a global health concern that has been categorized as a pandemic disease by the World Health Organization [2]. From 30 million in 1985 to 135 million in 1995, the number of people with diabetes mellitus is expected to rise to 541 million by 2030 [3]. This growing worry is especially evident in Palestine, where the Ministry of Health (MoH) says that the prevalence of type 2 diabetes (T2DM) is underreported, despite the fact that the incidence of DM in the Gaza Strip is 149.4/100.000 population. In 2014, diabetes accounted for 8.9% of all deaths, making it the fourth leading cause of mortality, according to the Palestinian Ministry of Health [4].

If left untreated, diabetes mellitus (DM) can have substantial long-term effects, particularly on dental health. According to research, patients with type 2 diabetes had a 29% and 22% higher chance of developing periodontitis and bone loss, respectively [5]. Additionally, people with uncontrolled diabetes have a threefold increased risk of developing periodontal tissue loss [6]. Diabetes affects many aspects of oral health, such as the function of the salivary glands, the brain, periodontal tissues, and the oral mucosa [7]. People with diabetes who have uncontrolled blood glucose levels frequently have dental issues. In those with diabetes, xerostomia (dry mouth) increases the risk of dental caries, oral fungal infections (oral candidiasis), poor wound healing, taste impairment, burning mouth syndrome, and periodontal disease [8].

In light of these concerns, it is critical that individuals with diabetes comprehend their increased vulnerability to oral difficulties, such as dry mouth, periodontal diseases, and dental caries, and the significance of preventing these issues with efficient treatment techniques. DM patients should be informed about the reciprocal association between oral health and DM to prevent and cure periodontal/oral disease and manage DM. Therefore, preserving periodontal health and proper dental hygiene helps diabetics maintain appropriate glycaemic control [9]. It is crucial to examine the knowledge and attitudes of patients with diabetes mellitus to determine their desire to adopt excellent oral hygiene habits. According to academic studies, promoting appropriate oral self-care habits requires increasing oral health knowledge [10]. However, everyday practice does not often follow from only knowing about and having a healthy attitude toward diabetes control and oral hygiene [11].

In Palestine, the primary health care services are mainly delivered by the United Nations Relief and Works Agency for Palestine Refugees (UNRWA) for registered refugees and by the MoH. Evidence showed that diabetes patients attending UNRWA clinics have high levels of chronic periodontitis and DMFT index and are less aware about diabetic complications on oral health [12, 13].Despite the growing number of DM patients in Palestine, little is known about oral health-related knowledge, attitudes, and behaviors (KAP) for patients attending primary health centers (PHCs) of the MoH. In order to create baseline data and, consequently, strategies for oral health awareness initiatives among patients with DM, it is required to evaluate the KAP of patients with DM with reference to oral health. This study sought to determine how well-informed T2DM patients were aware about the effects of diabetes on oral health, how they perceived oral complications and problems, how they described their oral hygiene habits, and to explore patients'experience and care preferences related to oral health**.**

Knowledge of common oral complications in different populations helps identify risk factors early. This allows for targeted interventions to prevent or manage oral diseases more effectively. Awareness of common attitudes toward oral health can help healthcare providers encourage better preventive practices. By considering patients'experiences and preferences, healthcare providers can create treatment plans that align with patients'expectations and comfort levels. This can increase patient satisfaction and adherence to prescribed oral health practices. Moreover, it leads to the development of policies and strategies aimed at improving oral health for underserved or at-risk groups. Understanding the specific oral health challenges faced by patients allows healthcare providers to offer better care tailored to their needs. In addition, Oral health is closely linked to overall health, and studying the interconnection between attitudes, practices, and complications helps to view oral health in a broader context. It supports the idea that maintaining good oral health is an essential part of overall well-being, which can improve long-term health outcomes. By understanding these aspects, healthcare systems can adapt to meet the needs of diverse populations, ensuring that everyone has access to quality oral health care and the knowledge to maintain it.

## Materials and methods

### Study design

A convergent mixed method design was conducted from May to November 2022. Quantitative and qualitative approach were used simultaneously. In the quantitative study, a questionnaire-based cross-sectional design was applied, while face to face interview-based study was followed for the qualitative study.

### Study setting

Gaza strip is divided into five governorates named in order from north to south: North, Gaza city, Middle Zone, Khanyounis and Rafah. The MoH in Gaza operates and runs 52 PHCs distributed in five governorates. Stratified simple random sampling was approached to select one PHC from each governorate at random based. These PHCs are ordered from north to south: Beit Lahia Martyrs, Al-Remal Martyrs health center, Der-Albalh Martyrs clinic, Khanyounes Martyrs clinic and Rafah Martyrs clinic.

### Sample size and sampling technique

The study population included all diabetic mellitus patients fulfilling the below mentioned inclusion criteria and those who visited the health care center during this study period. The online Survey Monkey website, available at https://www.surveymonkey.com/mp/sample-size-calculator/. was used to calculate sample size according to the last report of MoH (2021) which showed that 17.6% is the prevalence of T2DM, giving approximately a total of 11088 individuals above 40 years are registered within MoH PHCs. Sample calculation was done at 5% error and 95% confidence interval and revealed 372 patients should be included. The registered T2DM are distributed in the five governorates as follow: 1389, 5949, 918, 2173 and 689 individuals in the north, Gaza, middle area, Khanyounes and Rafah governorate, respectively. 

Random selection of participants following proportionate systematic sampling. The sample size included questionnaires from 376 T2DM patients. According to distribution of the 11,088 patients within the five governorates, the sample size was proportionally distributed: 48 from the north, 199 from Gaza, 31 from the middle zone, 73 and 25 from Khanyounes and Rafah, respectively. The distribution percentages are 12.5%, 53.5%, 8.25%, 19.5%, and 6.2%, in that order. Then, a list of females and males'patients was prepared from each PHC included in the study. The K^th^ interval was 30, which means that every 30 patients, from each list, come first to the PHC one is selected if he/she met the inclusion criteria. Regarding qualitative study, the two mains central PHCs, Gaza and Khanyouns martyrs, were chosen for doing interviews.

### Inclusion criteria

Patients who were diagnosed with diabetes mellitus and undergoing treatment for the same. In addition, patients were diagnosed with diabetes mellitus for at least five years and were willing to participate in the study.

### Exclusion criteria

Patients with mental health problems, hearing impairments, patients who were unable to provide the appropriate information and those not willing to participate in the study.

### Data collection procedure

A questionnaire, specifically designed for this study consisting of four parts has been developed to assess: 1) the awareness on oral implications of DM (17 questions), 2) oral hygiene practices of diabetic patients (15 questions), and 3) perception toward oral complications based on health belief model (HBM) (19 questions); perceived susceptibility to oral complications (5 questions), perceived severity of oral complications (6 questions), perceived benefits from oral hygiene (4 questions) and perceived barriers to oral hygiene (4 questions), in addition to the corresponding socio-demographic and clinical data (supplement 1). The questionnaire was prepared first in English, after reviewing many literatures found in the PubMed, and then translated into Arabic. The steps guidelines of the AHRQ were followed to translate and validate the questionnaire [14]. Forward backward translation was applied, face and content validity, in addition to internal reliability and test retest were determined accordingly.

Face validity was examined in terms of the clarity of the wording, the likelihood the target audience would be able to answer the questions, and the layout and style. Reviewers stated that the general shape of the questionnaire was organized and well arranged. Moreover, the questions were clear and easy to understand. For content validity, the content validity index -average approach (CVI/Ave) and Kappa statistics were calculated accordingly after the questionnaire being reviewed by nine referees. The item content validity index (I-CVI) and scale content validity index (S-CVI) ranged from 0.78 to 1 and 0.89 to 1, respectively. Kappa statistic (k*) for questionnaire’s items ranged from 0.79 to 1.00. The alpha cronbach (α) of awareness questions, HBM and oral health practices was 0.82, 0.87 and 0.84, respectively. The test re-test reliability, measured by ICC and Pearson correlation, ranged between 0.90 to 0.99 and 0.94 to 0.99, respectively.

Questions of awareness were measured on three points Likert scale (yes, no, don’t know), and questions of the HBM were measured on five points Likert scale (1=strongly disagree, 2=disagree, 3=neutral, 4=agree and 5=strongly agree). Moreover, similar Likert scale was used for questions of oral hygiene practices (1=never, 2=rare, 3=sometimes, 4=often and 5=always).

A semi-structured interview guide was developed to achieve the study objectives (13 questions with probing questions). Interview guide was presented to three health experts and dentists who have public health experience to ensure trustworthiness of questions, and to answer research questions. Moreover, interview guide was tested on two patients to make sure that questions are easy to be posed and understand. Patients were purposefully selected and we determined ten patients for the interview, but we decided to continue until saturation of information is achieved. At end, thirteen patients participated. A variety of ages and gender were represented in the sample of participants from the two centrals PHCs; Gaza and Khanyounis. We excluded patients who had not seen a dentist in the previous twelve months because our goal was to learn more about their experiences with dental professionals regarding diabetes. To ensure free bias selection of interviewees, the first thirteen individuals met the study criteria were asked to participate in the study, after the quantitative part is done, and if anyone refused, the invitation is given to next one. Interviews were conducted in a separate room within the PHCs. Audio records were transcript into papers.

### Data analysis

Data coding and analysis were done using the Statistical Package for Social Sciences software (SPSS) for Windows (version 26: IBM Corp). Prior analysis, data were checked for missing and extreme values and if a questionnaire has missing values of 5% or more, the questionnaire was discarded and not used for analysis. In descriptive analysis, means and standard deviation were used for analysis of continuous variables (age, duration of DM…), however, frequency and percentages were used for categorical variables (gender, level of education…), and the questionnaire items. In addition, mean percentages were presented for each item and for the whole domain (awareness, perception and practices). Perceived susceptibility to, severity of oral complications and benefits of and barriers to oral health habits are presented in graphs, and responses are presented in percentages into three categories; agree (agree and strongly agree), neutral, and disagree (disagree and strongly disagree).

About qualitative study, thematic analysis was approached. Codes were determined through reading line by line and codes which share similar ideas were pooled under one domain presented under themes.

## Results

### Socio-demographic and clinical characteristics of participated patients

The mean age for participants is 57.8 ± 8.76 years. Females represent 55.1% of the sample. Age is divided into five categories and patients below 55 and above 65 years constitute, in each, at least one fifth of the sample size. Half of the patients participated are from Gaza city (52.9%. 199/376) and the least percentage is from Rafah in the far south (6.6%). Majority of participants had formal school education (93.6%, 352/376), whereas 6.4% (24/376) are illiterate and 18.6% (70/376) had university degree. Most participants are not working at the time of data collection (72.9%, 274/376), and live under a deep poverty line (83.5%, 314/376). The mean duration of T2DM is 10.99 ± 6.61 years. Almost two thirds (64.2%, 241/376) have T2DM less than 10 years. The mean HbA1c value is 8.11% ± 1.84, and 62% and 38% have uncontrolled and controlled T2DM, respectively. Two thirds of participants have other chronic diseases in addition to DM, mostly hypertension (43.9%, 165/376) and hypertension with heart disease (10.9%, 41/376). Half (49.5%, 186/376) have family history of oral complications resulting from T2DM and 79.8% (300/376) of patients report oral manifestations in the last 12 months, and 56.6% (213/376) visited dental clinics accordingly. Common oral manifestations reported by participants are tooth decay (58.2%, 219/376) and tooth loss (53.2%, 200/376). Almost 30% (112/376) and 18.4% (68/376) suffer from gingival bleeding and bad odor, respectively. Reasons for visiting dental clinics are due to problems with teeth, gum or mouth (57.2%, 215/376) (Table 1).

**Table 1:** Socio-demographic and clinical information of participated patients (*n* = 376)

	Frequency	Percent	M ± SD
Age group			57.86 ± 8.76
50 or less	84	22.3	
51–55	69	18.4	
56–60	75	19.9	
61–65	70	18.6	
More than 65	78	20.7	
Residency			
North Gaza	48	12.8	
Gaza city	199	52.9	
Middle zone	31	8.2	
Khan Younis	73	19.4	
Rafah	25	6.6	
Are you smoker (number cigarette/days)			11.45 ± 8.28
Yes	45	12.0	
No	292	77.7	
Ex-smoker	39	10.4	
How many shisha	38	10.1	7.24 ± 3.26
Level of education			
Illiterate	24	6.4	
Up to elementary school	65	17.3	
Up to preparatory school	104	27.7	
Up to secondary school	113	30.1	
University and above	70	18.6	
Marital status			
Unmarried	62	16.5	
Married	314	83.5	
Income			
Under poverty line (< 1974 NIS)	314	83.5	
Above poverty line (≥ 1974 NIS)	62	16.5	
Number of family member			1.71 ± 0.76
5 or less	102	27.1	
6–10	219	58.2	
More than 10	55	14.6	
Working status			
Working	102	27.1	
Not working	274	72.9	
	Frequency	Percent	M ± SD
Having medical insurance			
Yes	367	97.6	
No	9	2.4	
Duration DM			10.99 ± 6.61
10 or less	241	64.1	
More than 10	135	35.9	
Family history of oral complications from T2DM			
Yes	186	49.5	
No	190	50.5	
Type of treatment			
Diet restriction and physical activity	18	4.8	
insulin injection	48	12.8	
Oral hypoglycemic agents	246	65.4	
Oral hypoglycemic agents and insulin injection	64	17.0	
Receiving education about oral care			
Yes	78	20.7	
No	298	79.3	
Last reading of HbA1c			10.27 ± 0.86
≥ 7%	143	38	
> 7%	233	62	
Presence other chronic diseases			
Yes	236	62.8	
No	140	37.2	
If yes, specify			
Hypertension	165	43.9	
Hypertension, Heart disease	41	10.9	
Heart disease	16	4.3	
Arthritis	4	1.1	
Asthma	4	1.1	
Hypertension, Asthma	2	0.5	
Arthritis, Hypertension	1	0.3	
Hypertension, cancer	1	0.3	
Hypertension, Kidney disease	1	0.3	
Osteoporosis	1	0.3	
Suffering from oral diseases in the last year			
Yes	300	79.8	
No	76	20.2	
Among yes reporting to have oral diseases in the last year			
Tooth mobility			
Yes	86	22.9	
No	290	77.1	
Tooth loss			
Yes	200	53.2	
No	176	46.8	
Tooth decay			
Yes	219	58.2	
No	157	41.8	
Tooth sensitivity			
Yes	24	6.4	
No	352	93.6	

*M* mean, *SD* standard deviation

Seven women and six men, ages ranging from 47 to 72, took part in the qualitative sample. Four themes were developed based on the extraction of fourteen codes: Dental care service quality, the patient-dentist interaction, oral hygiene and self-care, and the patient's experiences with oral health problems (Table 2).

**Table 2:** Codes and themes of qualitative study

Themes	Codes
Quality of dental care services	Accessibility
	Availability of essential dental services
	Affordability to cost
	Lack of dental
	Lack of medical supplies
	Appointment at PHC
	Follow up of dental status
Patient dentist relationship	Communication
	Health education and information
Self-oral care and hygiene	Awareness
	Attitudes
	Oral health practices
Patients’ oral health experiences	Dental problems
	Nutritional problem

### Patients'perception about oral complications from T2DM

Perceived susceptibility to oral health complications.

**Fig. 1:**
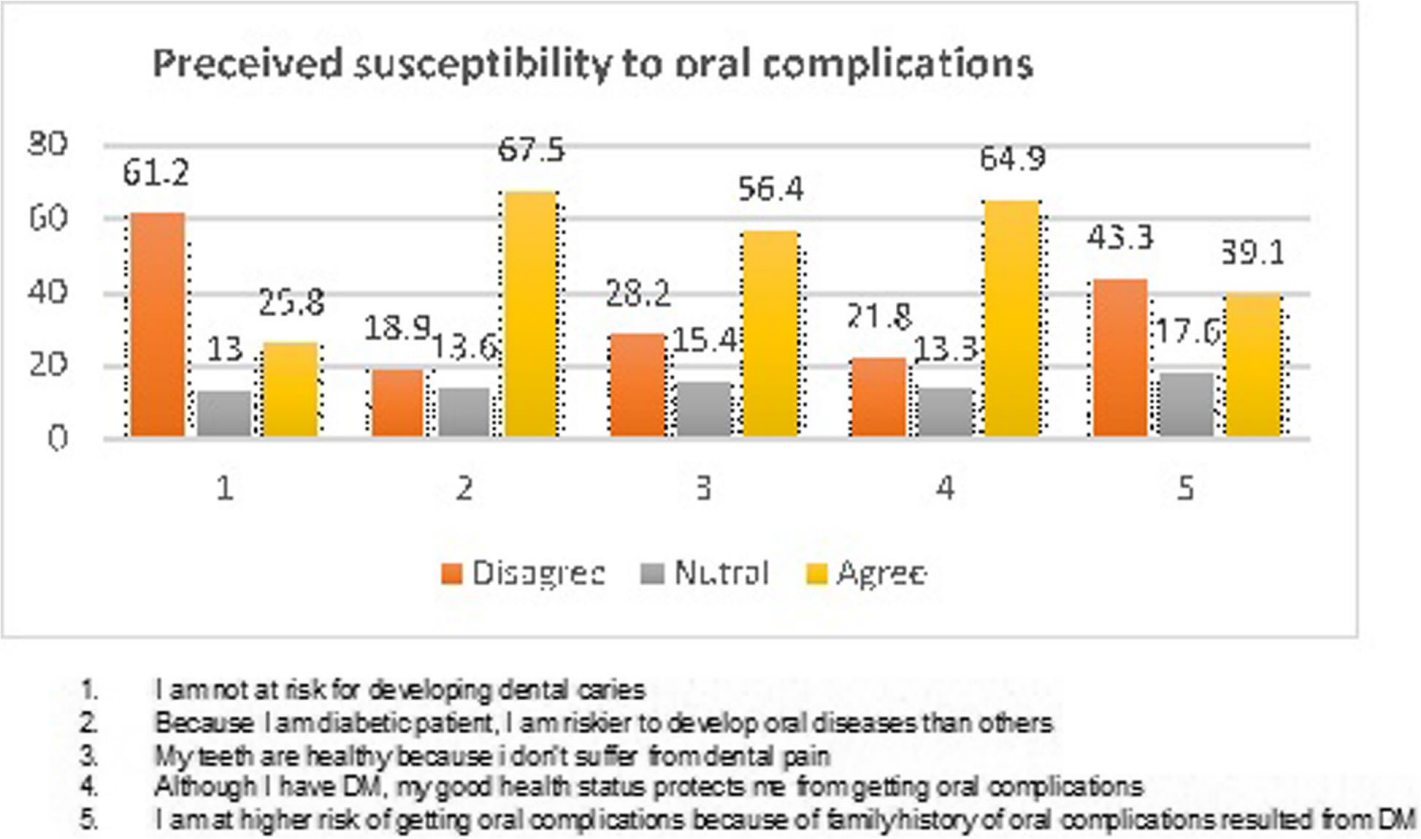
Perceived susceptibility to oral complications

On a five-point Likert scale, the mean score for perceived susceptibility to oral problems is 3.22 (0.32), indicating that 64% of patients believe they are at risk for oral complications. In particular, 59.2% of people with diabetes mellitus believe that they are more likely to experience complications than others due to their condition. The assertion"I am not at risk for developing dental carries"is also disputed by nearly two thirds (61.2%) (Fig. 1).

Perceived severity to oral health complications.

**Fig. 2:**
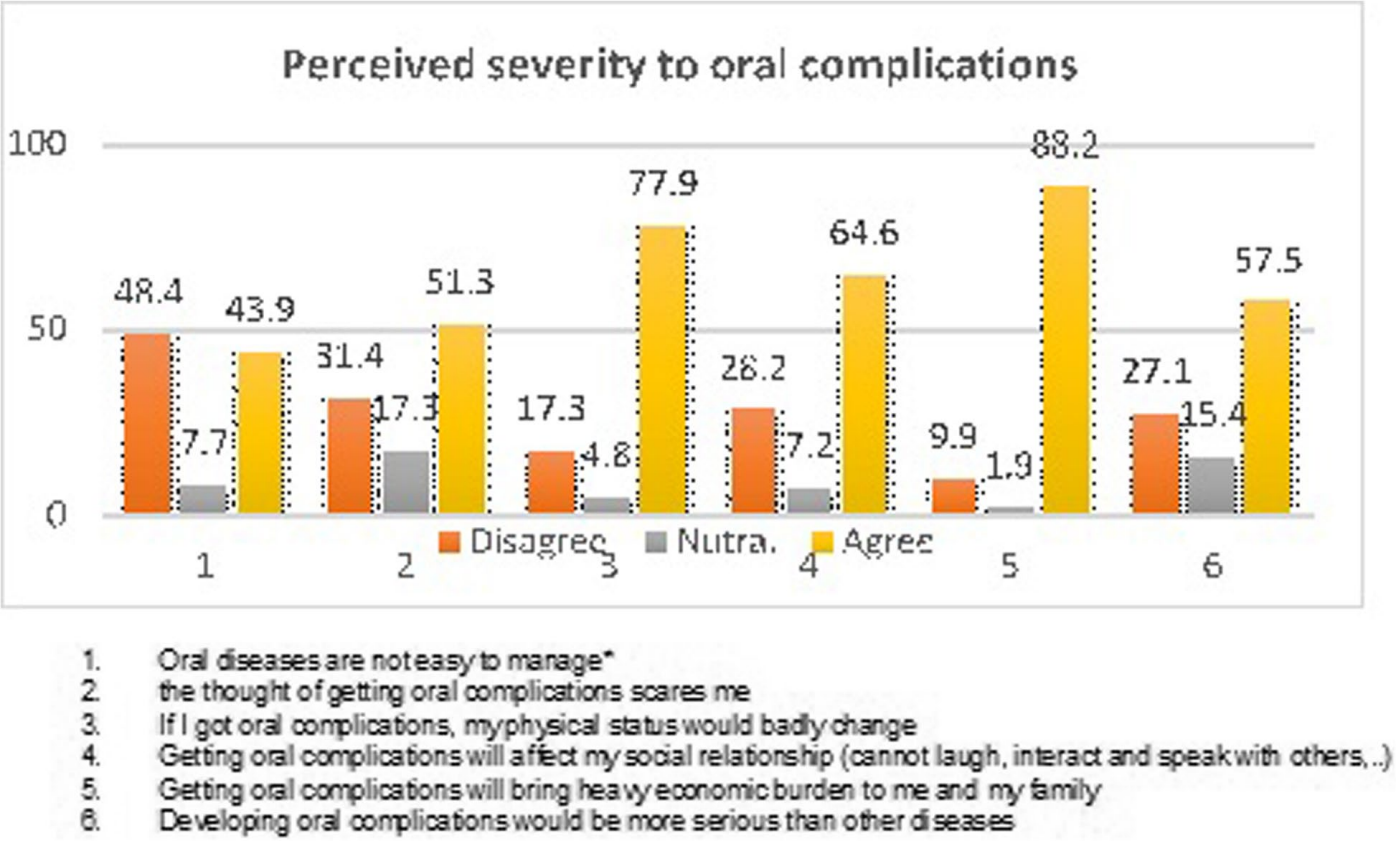
Perceived severity to oral complications

Most patients (67.8%) believe that oral problems are severe. On a five-point Likert scale, the mean score (standard deviation) is 3.39 (0.45). Most people (79.4%) think that dental issues have a big financial impact on patients and their families. Because oral problems negatively impact patients'physical status, 67% of respondents think they are more serious than other disorders (74.4%). In exchange, 58.6% of participants said that managing oral problems is simple (Fig. 2).

Perceived benefits from oral health practices.

**Fig. 3:**
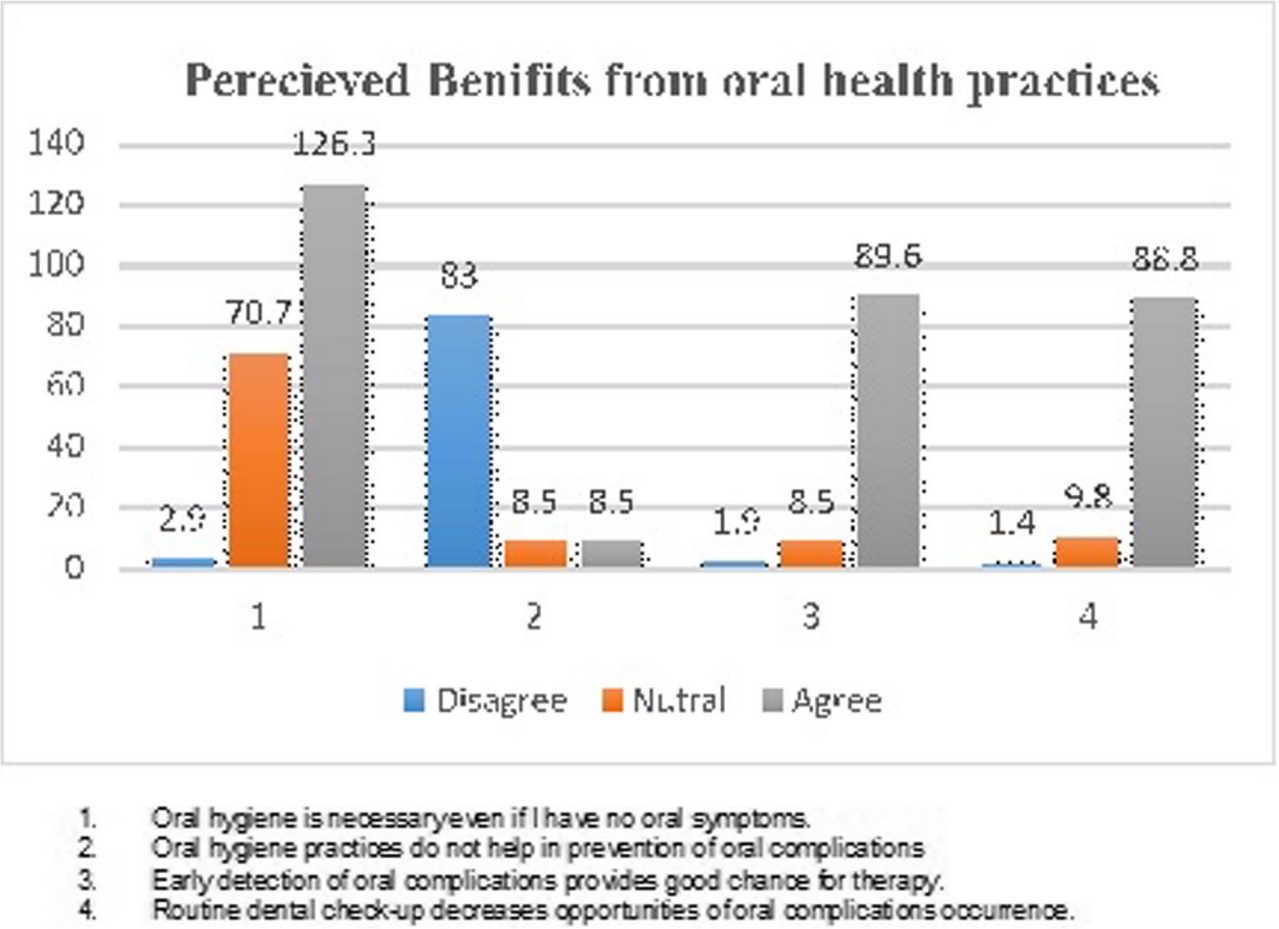
Perceived benefits from oral health practices

On a five-point Likert scale, the mean score for perceived benefits from oral health practices is 3.66 (0.36), indicating that 73.2% of respondents think that oral health practices provide advantages. In particular, 84.6% believe that maintaining good oral hygiene is essential even in the absence of mouth symptoms. Additionally, 80.6% believe that there is a good probability of therapy when oral symptoms are detected early. Only 56% of respondents, however, concur that maintaining good oral hygiene has little bearing on averting oral problems (Fig. 3).

The majority of interviewed patients do believe that keeping the mouth, teeth, and oral cavity clean is crucial to their overall health, as well as have favorable opinions on the value of follow-up in dental offices.

“…commitment with cleaning tooth has a good effect on health …” (Male, Khanyounis, 58 years)

“…in last 4 months I have a bad breath, I came to treat it, and the dentist prescribed me good mouth wash, then I felt good…” (Male, Khanyounis, 63 years)

“…I went to check my teeth because I know the effect of diabetic disease on my oral health…” (Female, Gaza,63 years)

“…brushing tooth daily with good nutrition is very important…” (Female, Gaza, 60 years)

“…we should brush our teeth to prevent gum inflammation …” (Female, Gaza, 51 years)

#### Perceived barriers to oral health practice

**Fig. 4:**
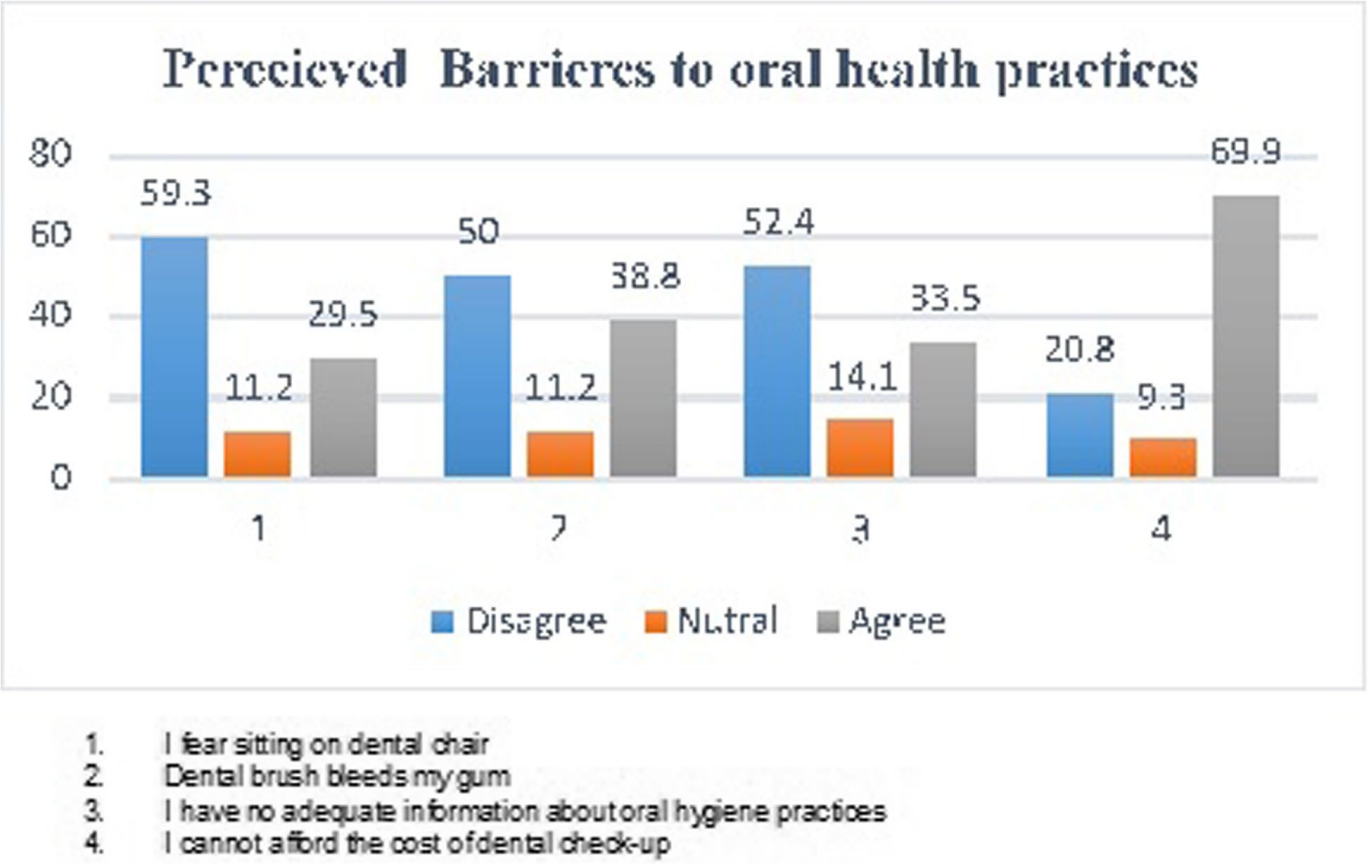
Perceived barriers to oral health practices

The average score on a five-point Likert scale is 2.81 (0.28). Many obstacles to oral health behaviors were perceived by more than half of individuals (56,2%). The inability to pay for a dental examination is the main reason why people avoid receiving oral health care (70.8%). However, half of the participants reported feeling that their gums bleed when they clean their teeth (54%), and they are afraid to sit in a dentist chair (57.6%). Furthermore, 55.4% of respondents said they don't know enough about proper dental hygiene procedures. Low practices are a result of these variables (Fig. 4).

Patients who were interviewed mentioned numerous obstacles, which can be categorized into variables linked to the institution, the doctor, and the patient. In reference to facility-related issues, interviewees expressed dissatisfaction with the inadequate dental services provided by governmental PHCs and the inability to obtain dental treatment or follow-up care when they visited a dental clinic because of a shortage of dental and occasionally medical supplies. Tooth extraction is the extent of dental services. Root canal therapy, tooth restoration, and scaling all necessary dental procedures for diabetic patients to maintain healthy gums and teeth are not available. One participant made it apparent that the lack of dental services and supplies results in poor care quality. Furthermore, basic dental and general practice supplies like gloves and sutures are not readily available, therefore occasionally dentists ask us to buy them from private pharmacies.

“…When you want to do restoration, they said it is finished and when you want to do root canal treatment, also they said has not existed, they just do tooth extraction …” (Male, Gaza, 47 years).

“…They haven’t anything to offer, bad dental care…” (Female, Khanyounis, 72 years) “… I'm taking two taxis to tell me it's finished, go back …” (Male, Khanyounis, 58 years)

“… I don't come here too much for treatment because they have nothing to offer…” (Female, Khanyounis, 72 years)

“…They told me there isn’t gloves to treat me…” (Male, Khanyounis, 58 years)

In terms of patient-related issues, the majority of patients interviewed reported dissatisfaction with the dental care provided by the governmental PHCs and having a challenging financial situation. They claim that because dental care is not properly offered by the PHCS, they cannot afford the prices at private clinics. The majority of them require artificial teeth to replace their extracted ones. Additionally, they stated that basic dental care, such as mouthwash, is not offered in PHCs and is expensive because we require it for an extended period.

“…. Expensive (treatments) when you want to rinse your mouth for 16 shekels every time and use an expensive antibiotic….” (Female, Gaza, 63 years)

“…. I can’t pay for dental treatment at private clinics ….” (Male, Khanyounis, 58 years; Male, Khanyounis, 63 years; Female, Khanyounis, 72 years; Male, Khanyounis, 57 years)

“…Root canal treatment at private sector is expensive I can’t pay for it, hope it found at governmental health care ….” (Female, Gaza, 51 years; Female, Gaza, 60 years)

### Awareness about oral health complications related to T2DM

According to Table 3, more than half of individuals are aware of the issue and provide accurate replies regarding oral difficulties brought on by type 2 diabetes. Most people (91%) agree that"diabetic patients require to care more often than non-diabetics for their teeth and mouth"(93.6%), and they also affirm the necessity of routine dental visits. The statement"Diabetics have gum problems more often if their blood sugar is uncontrolled"is met with an incorrect response from more than one-third (36.4%) of respondents. Regarding oral signs of type 2 diabetes, the responses of the patients who took part varied. The majority of patients are unaware that T2DM may cause taste issues and an oral burning sensation (74.2% and 73.7%, respectively). Furthermore, about 60% of patients are unaware that type 2 diabetes causes mouth ulcers, foul breath, and bacterial and fungal infections in the mouth.

**Table 3:** Awareness about oral health complications related to T2DM (*n* = 376)

Awareness on oral health complications related to T2DM items	Correct		Incorrect	
	n	%	n	%
Diabetic patients require to care more often than non-diabetics for their teeth and mouth	352	93.6	24	6.4
Regular dental visit is more important for diabetic patients than non-diabetics	342	91	34	9
Diabetics have gum problems more often if their blood sugar is uncontrolled	239	63.6	137	36.4
Oral problems associated with diabetes (prompted):				
Mouth dryness	304	80.9	72	19.1
Gums bleeding on brushing•	185	49.2	191	50.8
Mouth ulcers	152	40.4	224	59.6
Bad odor	153	40.7	223	59.3
Dental caries	279	74.2	97	25.8
Oral bacterial infections	142	37.8	234	62.2
Fungal mouth infections	157	41.8	219	58.2
Loose teeth	308	81.9	68	18.1
Taste problems	97	25.8	279	74.2
Burning sensation	99	26.3	277	73.7
Smoker diabetics have less serious gum disease than non-smokers. *	304	80.9	72	19.1
T2DM causes delayed wound healing at dental extraction sites	282	75	94	25
T2DM contributes more to developed dental abscess	165	43.9	211	56.1
as a diabetic, poor oral health condition affects badly the general health status	339	90.2	37	9.8
Total	57.61		42.39	

*a reverse question

*M* mean, *SD* standard deviation

Few (2/13) respondents to the qualitative study disagreed with the link between type 2 diabetes and oral complications, indicating that DM has no effect on teeth. In exchange, the majority (11/13) concurred dental issues brought on by diabetes mellitus and how it affects not just teeth and oral cavities but also other systems.

“Diabetic does not affect teeth, there is no relation between them” (Male, Khanyounis, 64 years).

“Sure, diabetic disease affect my teeth and nerves and everything at my body…” (Male, Khanyounis, 57 years)

“There is direct relationship between diabetic disease and teeth problem, diabetic make bad odor for mouth” (Male, Khanyounis, 63 years)

“High glucose affects my eyes, teeth and even my effort, it affects everything…” (Female, Gaza, 51 years)

### Oral health practices

On a five-point Likert scale, the mean score for oral health practices is 1.71 (0.17), meaning that just 42.5% of respondents are dedicated to providing the greatest oral hygiene treatment. The majority of patients use a toothbrush and paste to clean their teeth and mouth (81% and 79.5%, respectively). On the other hand, 61.75% of people clean their teeth for at least two minutes each time, and 58.25% do so twice a day. One-third (32%) do go to the dentist twice a year for a checkup, and two-thirds (68.5%) change their toothbrush four times a year (Table 4).

**Table 4:** Distribution of participants'responses regarding oral health practices (*n* = 376)

Oral health practices items	Never n(%)	Rare n(%)	Sometime n(%)	Often n(%)	Always n(%)	M	SD	% mean
I brush my teeth twice a day	67 (17.8)	62 (16.5)	84 (22.3)	69 (18.4)	94 (25)	2.33	1.34	58.25
I brush my teeth for at least two minutes each time	62 (16.5)	44 (11.7)	77 (20.5)	84 (22.3)	109 (29)	2.47	1.35	61.75
I use toothbrush and paste to clean my teeth	25 (6.6)	24 (6.4)	61 (16.2)	37 (9.9)	229 (60.9)	3.18	1.18	79.50
I follow the vertical technique of brushing the teeth	66 (17.6)	35 (9.3)	101 (26.9)	52 (13.8)	122 (32.4)	2.44	1.40	61.00
I used to change toothbrush four times a year	50 (13.3)	40 (10.6)	76 (20.3)	41 (10.9)	169 (44.9)	2.74	1.38	68.50
I use mouthwash to preserve and keep my gum healthy	215 (57.2)	81 (21.5)	46 (12.2)	15 (4)	19 (5.1)	1.00	1.24	25.00
I remove interdental debris using dental floss or toothpick once a day	167 (44.4)	89 (23.7)	52 (13.8)	23 (6.1)	45 (12)	1.41	1.41	35.25
I clean my tongue	224 (59.6)	39 (10.4)	59 (15.7)	16 (4.2)	38 (10.1)	1.05	1.39	26.25
Using Silica Powder for tooth cleaning	338 (89.9)	18 (4.8)	12 (3.1)	1 (0.3)	7 (1.9)	0.24	0.76	6.00
Using Miswak for tooth cleaning	224 (59.6)	69 (18.4)	54 (14.3)	14 (3.7)	15 (4)	0.93	1.20	23.25
Using finger and paste for tooth cleaning	286 (76.1)	19 (5.1)	43 (11.4)	17 (4.5)	11 (2.9)	0.58	1.09	14.50
Using rinse with salty water for tooth cleaning	105 (27.9)	51 (13.6)	110 (29.2)	63 (16.8)	47 (12.5)	1.86	1.33	46.50
Using rinse with herbs for tooth cleaning	236 (62.8)	50 (13.3)	53 (14.1)	17 (4.5)	20 (5.3)	0.90	1.25	22.50
Using rinse with water only for tooth cleaning	15 (4)	9 (2.4)	79 (21)	50 (13.3)	223 (59.3)	3.24	1.06	81.00
I used to visit a dentist, for checkup, twice a year	180 (47.9)	94 (25)	45 (12)	25 (6.6)	32 (8.5)	1.28	1.34	2.00
Total						1.71	0.52	42.5

*M* mean, *SD* standard deviation

Most interviewed patients do brush their teeth with toothpaste twice daily. Few used medical mouth wash, however based on medical advice. Others have used salty water to relief pain from gums. One patient stated that brushing teeth is based on his mood status.

“…I rinse my mouth every day 3 times and brush my teeth after meal and before bed…” (Male, Khanyounis, 63 years)

“…I just clean my teeth with water…” (Female, Khanyounis, 72 years)

### Oral health problems

According to the qualitative study, the majority of patients who were interviewed had a variety of gum and dental issues, such as loss, mobility, and dental carries. Few patients did not have oral and dental issues, and five patients lost all of their teeth. Only two patients reported having ulcers and foul breath.

“…I haven’t any teeth at my mouth, all are falling…” (Male, Khanyounis,58 years; Female, Khanyounis,72 years; Male, Khanyounis, 64 years)

“…my teeth were prosthetic, all are bad…” (Male, Khanyounis, 63 years)

“…from two months ago, I went to dentist, and he said to me that your teeth are good…” (Female, Gaza, 63 years)

“…most of my teeth are mobile …” (Female, Khanyounis, 54 years)

“…my teeth and gum are good, and I just need a restoration…” (Male, Gaza, 47 years)

“…I extracted more than one tooth after I am diagnosed with diabetic disease …” (Female, Gaza, 60 years)

The inability to chew food is a common complaint among patients with gum and dental issues. Additionally, they have been denied access to all the things they used to consume before to the onset of oral difficulties. Some of them believe that since sugar and carbohydrate products deteriorate their oral and physical health, their nutritional condition needs to be corrected and adjusted by consuming fewer of them. For two of them, diabetes has no impact on their nutritional health.

“…when you avoid eating sugar, your teeth and health will be good…” (Female, Gaza, 51 years)

“…diabetic disease didn’t affect my nutrition and my teeth…” (Female, Khanyounis, 54 years)

“…I face difficulty with chewing food …” (Male, Khanyounis,58 years; Female, Gaza, 50 years)

“…I feel sad when I look to my teeth, all are fallen and then I can’t eat…” (Female, Khanyouunis, 72 years)

“…my nutrition system is change, I stopped eating sugary and chocolate…” (Female, Gaza, 60 years)

### Patients’ experiences

For patients with diabetes, patient follow-up is crucial. In fact, the majority of patients interviewed stated that they are dissatisfied with the follow-up system that the dentists give and that PHC dental clinics lack an appointment system. Furthermore, if there are dentists at their place of employment, they do not schedule follow-up appointments.

"…There is no follow up but only extraction (tooth), and if you need management they say (dentists) do it outside…"(Male, Khanyounis, 58 years)

"…I rarely follow at PHCs, I follow mainly in private clinics…"(Female, Khanyounis,54 years)

“…no one (doctors at PHCs) have asked me about my teeth or referred me to dentist for checkup…” (Female, Gaza, 50 years)

Many patients complained about poor time management in between appointments, long wait times, and the fact that dentists only checked their teeth briefly before referring them to private clinics for further dental care.

“…I don’t like to take turn (in governmental dental clinic), I came just when I am so tired from dental pain…” (Female, Gaza, 63 years)

“…you should go so early to get number for your turn ….” (Male, Gaza, 47 years) “…Dentist doesn’t want to work or even treat us….” (Female, Gaza, 50 years)

Respondents expressed discontent with the actions of dentists. They expressed dissatisfaction with the lack of information provided by dentists regarding how to appropriately treat oral issues. Additionally, glycemic level and medical status are not inquired about by dentists. Even the impact of diabetes treatment on dental health is not covered. Communication with dentists is satisfactory for two participants.

“…they advise me how to brush and rinse my mouth and before they work, they ask me and care about me …” (Female, Gaza, 63 years)

“…they (dentists) didn’t give me any information…” (Male, Khanyounis,64; Female Khanyounis,72 years; Female, Gaza, 51 years; Male, Gaza, 47 years)

“…they (dentists) didn’t tell us how we should care…” (Male, Khanyounis, 58 years; Male, Khanyounis, 72 years; Male, Khanyounis, 64 years)

“…the care of dentist didn’t change when he know that I am diabetic, just he answers me when I asked…” (Male, Khanyounis, 63 years)

PHCs were criticized by patients because they lack established health education programs at this time, and because dentists either don't provide any information at all or supply it enough, patients mostly rely on other sources like websites, friends, or television to learn about the effects of diabetes mellitus on oral health.

“…I get my information about diabetic disease and its effect from T.V, internet and my neighbors…” (Female, Khanyounis, 54 years; Female, Gaza, 60 years)

“…they didn’t give us health promotion program, or any information related to our status…” (Male, Khanyounis, 58 years)

“…I always read about my disease from internet…” (Female, Gaza, 63 years)

“…we go, and they didn’t even talk to us, they just examine us and then say goodbye…” (Female, Gaza, 50 years)

## Discussion

The World Health Organization has declared diabetes mellitus (DM) a pandemic due to its sharp rise in prevalence worldwide. As a result, the bidirectional association between diabetes mellitus and dental health, particularly periodontitis, concerns medical practitioners, including dentists. Good knowledge and positive attitudes are recognized to have an impact on effective oral practices [10]. People with Type 2 Diabetes Mellitus require an assessment of their degree of awareness regarding the oral symptoms of the disease and its associated consequences on oral health in order to establish appropriate health care recommendations, patient education, and health care delivery to diabetic patients.

Patients in this study expressed favorable opinions on the risks and consequences of oral health issues, as well as the advantages and disadvantages of maintaining good oral hygiene. In Ethiopia, 25.5% of participants believed that periodontal disease could lead to heart disease, and 18.8% agreed that a person with diabetes has a higher risk of developing periodontal disease [15]. 72% of Chinese people also believed that they were at risk for diabetic complications, such as oral manifestations, and its risk factors [16]. The majority of Turkish patients were aware of their vulnerability to oral problems from diabetes mellitus, such as dry mouth, tooth decay, bad breath, and bleeding gums. Other problems, such as fungal infections, taste impairment, and oral ulcers, were less likely to occur, though [16]. Despite the severity of periodontitis itself, Abdullah and his colleagues found that patients have rated type 2 diabetes as more serious than the disease [17]. 64% of Ethiopian patients believed that their DM was the cause of their severe oral health issues [15]. In exchange, fewer patients expressed satisfaction with the degree of DM oral cavity problems, such as bleeding gums and tooth movement [18]. Studies from Saudi Arabia and Kuwait also reported the positive opinions [19, 20]. We did not study the link between smoking and periodontitis, although literature highlighted the direct effect of smoking on oral health especially periodontitis. We recommend further studies on this issue.

Sahile et al. [15] found that 53% of participants thought that practicing good dental hygiene would help them avoid developing periodontitis. In a different study that involved sizable groups of diabetic patients, the patients felt that maintaining good dental hygiene led to a significant decrease in gingival bleeding and mouth inflammation [21]. The literature has documented numerous obstacles. Dental expenses were the main reason why people avoided or postponed dental visits, according to Poudel et al. [22].

Furthermore, 71% and 70% of individuals, respectively, confirmed that they lacked insurance coverage and access to dental treatment [23]. In a similar vein, Fadel et al. [24] from Saudi Arabia documented differences in the frequency of dental visits and oral health practices, with the primary disadvantages being lengthy wait times and expensive treatment. In fact, dental and oral health practices were strongly predicted by self-efficacy and knowledge of the consequences of type 2 diabetes on oral health [25]. Inadequate training and follow-up by dentists were also addressed, along with worry and dread [26, 27]. According to a study by Cankaya et al. [17] in Turkey, medical practitioners don't give enough advice about dental health care. Additionally, practitioners did not adequately explain the significance of interdental cleaning, maintaining good oral health, brushing teeth, and oral examinations. We think that in busy practices, dentists may have limited time for each patient, leading to a focus on immediate concerns rather than comprehensive education. In addition, dentists might assume that patients already understand the importance of interdental cleaning and good oral hygiene, resulting in less emphasis on these topics. Some dentists may struggle with effectively communicating complex information in a way that is easily understood by patients, and also some patients may not actively engage in conversations about their oral health, leading dentists to focus on more pressing clinical issues instead.

We found 57.6% of participants are aware of how type 2 diabetes affects dental health. This was lower than the findings from Kuwait [18] and Iran [28], but it was almost identical to the findings from Egypt [29], Tanzania [20], and the USA [30] and Saudi Arabia [31]. The research also noted a lack of information about oral health [22]. Saudis had a low level of awareness, according to Ismail and Ali [29]. In exchange, 82.7% of participants knew that diabetic people require specialized medical care, and 63.4% of individuals knew that diabetes affects dental health [32]. In Saudi Arabia, diabetic patients are aware of the importance of controlling their diabetes in order to minimize oral health complications, but only a small percentage of them visit the dentist on a regular basis. Variations within the same country could be attributed to the weakness of their primary health facilities across the country and the limited lack of educational campaigns. The results of these different studies can be attributed to differences in methodology and the study population. Furthermore, many patients may not receive adequate education about the connection between diabetes and oral health during their diagnosis or ongoing treatment. Insufficient communication from healthcare providers, including dentists and diabetes educators, can lead to gaps in knowledge about oral health risks. Patients with lower health literacy may struggle to understand medical information, making it difficult to grasp the importance of oral health in diabetes management.

Similar to data from South Asia [33] and Tanzania [20], the majority of participants had a good attitude about oral health, although their oral health-related practices were low (42,5%). The results of this study do show that maintaining excellent dental health depends on a variety of factors, including practices linked to oral health, rather than only one's attitude. The literature found that DM patients'oral hygiene practices varied similarly. 58% of the patients in our study brushed their teeth twice a day, falling between the 24.2% and 95.3% range reported in the literature [17, 34]. Contrary to Cankaya and his colleagues'findings, one-third of our patients utilized toothpicks to remove interdental debris [17]. Despite the fact that some dentists advise against using toothpicks, many diabetic patients still using them for cleaning [35]. As a source of speculation, while patients may understand the importance of oral health, they might lack specific knowledge about effective practices, such as proper brushing techniques or the importance of interdental cleaning. Some diabetes patients may experience complications, such as neuropathy or mobility issues, making it physically challenging to maintain good oral hygiene practices.

The percentage of T2DM patients who visit the dentist on a regular basis varies greatly [17, 36]. Just 2% of T2DM patients in our study saw their dentists every six months. The socioeconomic condition of the patients that is, their degree of education and financial resources as well as aspects pertaining to the health facility may be the attributing factors for low dental clinic attendance [37, 38]. Patients with diabetes must see a dentist on a regular basis to prevent tooth pain and infections that could impair metabolic control, as well as to discover oral issues associated with the disease early. Patients in this study expressed dissatisfaction at the lack of an appointment system, which is in line with other reports [17].

We found that there are also deficiencies in the individuals who are referred to dentists by their attending physicians. A study from Tazania and Australia also found that people with diabetes mellitus are not often referred to dentists [20, 39]. The low number of referrals could mean that doctors are unaware of the strong link between diabetes mellitus and oral health, or that the health system lacks official clinical standards to assist doctors in choosing which dental specialists to recommend patients to. In another hand, high number of patients seen by physicians may influence patients to receive the right care including being seen by dentists.

According to numerous interviewers, the lack of education programs at PHCs may be the reason why patients are not well-informed about the complications of diabetes mellitus on dental health. Patients might, however, rely on other sources, including TV shows, the internet, and friends and family, which could be false or misleading. The majority of patients who were interviewed concurred that dental evaluations and appointments should be scheduled during medical visits. According to the literature, DM patients believe that doctors, nurses, and other healthcare professionals are ineffective at informing them about the oral problems of the disease [35].

In summary, the findings highlight significant gaps in patients'awareness and perceptions regarding oral health in the context of diabetes mellitus. While a majority acknowledge their susceptibility to oral health issues and recognize the severity of potential complications, a considerable percentage still perceive barriers that hinder effective oral health practices. The inadequate awareness of oral health complications and poor oral health habits suggest a need for enhanced educational initiatives and targeted interventions. The qualitative insights emphasize the importance of dental care service quality, effective patient-dentist interactions, and the promotion of self-care practices. Addressing these factors is essential to improve patient outcomes and foster better oral health practices among individuals with diabetes, ultimately leading to a reduction in oral health complications and an enhancement of overall well-being.

There are limitations to the study: 1) although the qualitative study had a modest number of participants, it reached saturation information and represented the two primary health center PHCs in the Gaza Strip. 2) the nature of cross-sectional studies which restrict the causal linkages between variables. The study's strength is that it's the first mixed-method design to use a qualitative approach to investigate patients'oral health experiences. Second, self-report responses are not subject to criticism because data collection was conducted through face to face interviews. Thirdly, conclusions drawn from this study could be used in other contexts. The study offered some metrics for the parameters under investigation and could be a useful tool for enhancing the oral health care of diabetics.

### Policy implications

1. Integration of oral health into diabetes management policies: national healthcare guidelines should formally integrate oral health assessments into routine diabetes care protocols because recognizing the bidirectional relationship between diabetes and oral health, especially periodontal disease, will help promote holistic management and early intervention
2. Development of targeted public health education campaigns: launching culturally sensitive and accessible educational initiatives focused on raising awareness of the link between diabetes and oral health. Addressing knowledge gaps and misperceptions can empower patients to engage in preventive behaviors and seek timely dental care.
3. Subsidized or covered dental services for patients with DM: expanding insurance coverage or provide subsidies for routine dental visits and preventive care for people with diabetes, because financial barriers were identified as a key perceived obstacle. Removing cost as a barrier can encourage regular dental check-ups and early treatment.
4. Inter-professional collaboration and training: implementing policies that encourage collaboration between medical and dental professionals, including cross-training on diabetes and oral health, as better communication and shared knowledge among providers enhance coordinated care and improve patient guidance.
5. Quality assurance standards for dental care providers: introducing quality assurance standards that include patient satisfaction, communication effectiveness, and diabetes-specific care protocols in dental practices. Evidences showed that improving patient-dentist interactions were emphasized as critical in the qualitative data; policies should support continuous provider education and accountability.

## Supplementary Information

Supplementary Material 1.

## Data Availability

The datasets used and/or analyzed during the current study are available from the corresponding author on reasonable request.
