# Supplementary material for: Exploring oral health related awareness, perceptions, practices and experiences among type 2 diabetes mellitus patients: a mixed method design
Source: BMC Oral Health. 2025 May 24;25:781. doi: 10.1186/s12903-025-06153-5 (PMC12103006; doi:10.1186/s12903-025-06153-5)
Supplement: Supplementary file 1 — Supplementary Material 1. [file 12903_2025_6153_MOESM1_ESM.docx]

**Oral Health Status Among Type II Diabetes Mellitus Patients Attending Governmental Primary Health Centers at Gaza Governorate**

**Sociodemographic characteristics** 1 **Serial number:** ( ) **Age**: ( ) years

Gaza

( ) ( ) ( )

**(** **)** Yes **(** **)** No


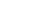


2 **Gender:** ( ) male ( ) female

( )

3 **Place** North Gaza city Mid zone Khanyuouns

( ) Yes How many

4 **Are you smoker:** cigarettes? ( ) ( ) No How many hookah?

( ) **Educational level:** o Illiterate

o Up to primary school

5 o Up to preparatory school o Up to secondary education o University or above

( ) Rafah

( ) Ex-smoker


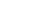

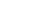

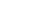

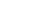


6

**Marital status:**

( )single ( ) married ( ) other

7 **Income :** …………..

8

9

10

11

12

12

**Number of family members :** ………………

**Workin** ( ) ( ) not ( ) ( ) free ( )

**g status:** employed employed Retired lancer Housewife **Do you have a**

**medical** ( ) Yes ( ) No **insurance?**

**Clinical characteristics**

**Diabetic age (How many years with T2DM?):** ………….……. Years

**Family history of oral complications from T2DM?**

**Type of treatment:** oDiet restriction and physical activity oInsulin injections


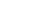

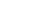

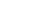


oOral hypoglycemic agents

oOral hypoglycemic and insulin injections


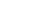


118

13


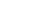

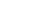


**Have you received health education regarding oral health practices**?

( ) Yes ( ) No

14 **The last value of HbA1C**? ………………………………………….

15

16

17

18

19

**The last value of fasting blood glucose level**:

( ) Yes **Do you have other** **Please specify:**

**chronic diseases?** ……………………………………… ……

( ) Yes Specify ;

( )Tooth mobility ( )Tooth loss **oral and dental diseases** ( )Tooth decay ( )Tooth **during the last year?** ( )Gum bleeding ( )Bad Odor

**Have you suffered from**

sensitivity

( )Bacterial infection ( )Fungal infection

**During the last year,**

**have you visited a dental** ( ) Yes **clinic?**

**What was the reason** o Consultation/advise

**for your last visit to the** o Pain or trouble with teeth, gums or mouth **dentist?** o Treatment/ follow-up treatment

o periodic check-up

o Don’t know/don’t remember

( ) No

( ) No

( ) No


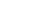

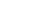

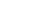


**Perceived susceptibility to oral Strongly complications Disagree**

I am not at risk for 20 developing dental

caries.*

Because I am diabetic patient, I am riskier to develop oral diseases than others.

21

My teeth are healthy 22 because i don’t suffer

from dental pain

Although I have DM, my good health status protects me from getting

23

oral complications.

I am at higher risk of getting oral complications because of family history of oral complications resulted from DM.

24

**Disagree Neutral Agree**

**Strongly Agree**

**Perceived severity of oral complications**

Oral diseases are not easy to manage*

25

The thought of getting 26 oral complications scares

me.

If I got oral complications, my physical status would badly change. Getting oral

27

complications will affect 28 my social relationship

(cannot laugh, interact and speak with others,..) Getting oral complications will bring heavy economic burden to me and my family. Developing oral complications would be more serious than other diseases

29

30

**Perceived benefits from oral health practices**

Oral hygiene is

31 necessary even if I have no oral symptoms.

Oral hygiene practices 32 do not help in prevention

of oral complications.* Early detection of oral

33 complications provides good chance for therapy. Routine dental checkup decreases opportunities of oral complications occurrence.

34

**Perceived barriers to oral health practices**

I fear sitting on dental chair

35

Dental brush bleeds my gum

36

I have no adequate

37 information about oral hygiene practices*

I cannot afford the cost of dental checkup*

38

**Strongly Disagree**

**Strongly Disagree**

**Strongly Disagree**

**Disagree**

**Disagree**

**Disagree**

**Neutral Agree**

**Neutral Agree**

**Neutral Agree**

**Strongly Agree**

**Strongly Agree**

**Strongly Agree**

120

**Awareness on oral health complications related to T2DM** Yes No Don't know

39 Diabetic patients require to care more often than non-diabetics for their teeth and mouth

40 Regular dental visit is more important for diabetic patients than non-diabetics.

41 Diabetics have gum problems more often if their blood sugar is uncontrolled

42 Oral problems associated with diabetes:

• Mouth dryness

• Gums bleeding on brushing

• Mouth ulcers

• Bad odor

• Dental caries

• Oral bacterial infections.

• Fungal mouth infections

• Loose teeth

• Taste problems

• Burning sensation

43 Smoker diabetics have less serious gum disease than non-smokers.*

44 T2DM causes delayed wound healing at dental extraction sites 45 T2DM contributes more to developed dental abscess

46 As a diabetic, poor oral health condition affects badly the general health status

**Oral health practices** **Never Rarely Sometimes** **Often** **Always** 47 I brush my teeth twice a

day

48 I brush my teeth for at least two minutes each time

49 I use toothbrush and paste to clean my teeth

50 I follow the vertical technique of brushing the teeth

121

51 I used to change toothbrush four times a year

52 I use mouthwash to preserve and keep my gum healthy

53 I remove interdental debris using dental floss or toothpick once a day

54 I clean my tongue

55 I use other methods than toothpaste and toothbrush to clean my mouth:

Silica Powder

Miswak

Finger and paste

Rinse with salty water

Rinse with herbs

Rinse with water only

56 I used to visit a dentist, for checkup, twice a year
